# Report of Discussions before the Ohio State Dental Society, Columbus, December 2d., 1874

**Published:** 1875-03

**Authors:** 


### Proceedings of Societies.

### REPORT OF DISCUSSIONS BEFORE THE OHIO STATE DENTAL SOCIETY, COLUMBUS, DECEMBER 2d., 1874.

Continued from page 92.

Dr. J. H. Warner: While the subject of Dr. Craven's discovery is before the house, I want to add my mite of information. Have had no experience in the use of lacto-phosphate of lime myself, but my brother has given it very careful consideration for two years. He was in correspondence with Dr. Craven at the time the discovery was made. He tested it thoroughly and abandoned the ordinary mode of practice for the method proposed by Dr. Taft. He says that the remedy had proved a failure in his hands until the time he adopted Dr. Taft's method, since when, it has proved a flattering success. He first cleanses the cavity and then protects the pulp with a preparation of carbolic acid and phosphate of lime. After it becomes somewhat hardened he fills the cavity with oxy-chloride of zinc, and finally completes the operation with gold. According to the reports made at the Detroit Convention, that process seems to have given the best results.

Dr. Taft: A little explanation may not be out of place.

Dr. Cravens gave a caution in reference to the liability of the lacto-phosphate of lime to decompose. It is then, of course entirely unfit for use. Many failures are doubtless due to the use of the remedy after it has begun to decompose.

For several months I have been in the habit of keeping the two ingredients, lactic acid and phosphate of lime, separate, until I have had occasion to use them. Have had as good success with the remedy as with anything else. Dr. Reed, of Cincinnati, came to me some three months ago with the right inferior wisdom tooth decayed on the buccal surface. It ached severely. There was a complication of inflammation of the pulp and extreme periostitis. Tried lacto-phosphate, but it seemed only to aggravate the difficulty. In this case it failed. It was a very unfavorable one. In such instances, unless the natural recuperative powers are remarkably good, the tooth will be lost.

Dr. Rehwinkle: I have met with failure in such cases.

Dr. Clayton: I am interested in this matter which has been presented, and particularly so, because it shows a recognition on the part of the profession, of the fact that there is something yet to be learned in regard to the treatment of exposed pulps.

It is important, as we have learned, to consider carefully systemic conditions. We generally find congestion of the pulp when there is much pain. This must be relieved. To accomplish it I use an astringent. Sometimes you will find that an astringent only makes matters worse by choking up the passsges. When that occurs and there is a state of con gestion accompanied by throbbing pain, I prefer to cut the matter short by depleting the pulp. Then apply carbolic acid. I wait awhile, and if I have succeeded in securing perfect immunity from pain,—say for twenty-four hours—I feel that the case is hopeful. I then proceed and use carbolic acid and oxide of zinc, and then fill over all with oxy-chloride of zinc mixed as thick as possible. When there is inflammation of the pulp, our treatment, of course, sometimes fails. Sometimes I find the pulp engorged and protruding through the orifice of exposure. I cut away the part protruding and apply carbolic acid in full strength for its escharotic effect.

I have succeeded in several cases which I treated in this way. If we extract a tooth which has ached a very little, we will find that there is a very limited surface of the pulp which is congested. This indicates that it is practicable to save a portion of the pulp by sacrificing the part which is diseased.

In cases of recent exposure I fill the cavity with clean cotton, and melt wax upon it. One of the causes of failure, I think, is a lack of judgment as to when we shall fill, or perhaps, what we shall fill with. The important point is to know *when* to fill.

Again it is important to inspire the patient with faith. We must, ourselves, have faith. There is sometimes a lack of sympathy between the patient and the operator which acts as a barrier. There are persons for whom we can not work with any degree of pleasure. There is a mutual feeling of repugnance.

Dr. Horton: I cannot expect to enlighten these older gentlemen upon this subject. I would make a suggestion however in regard to an application to be used preparatory to the treatment recommended by Dr. Taft. This is the formula: Take one to one and a half grains of aconitine, put in a phial with an ounce of creosote. Apply to the nerve on a small bit of cotton.

Dr. Porter: Dr. Wells remarked that it does not make so much difference when we fill as with what we fill. I think he might have added that it matters much *how* we fill.

It is greatly to be deplored that some men persist in treating the exposed nerve with escharotics. I do not believe in applying oxy-chloride of zinc to an exposed pulp. If you have a cavity which is sensitive merely, then apply it. But in cases of absolute exposure I would not in any case use it.

Some one has said that he does not know whether this preparation recommended by Dr. Cravens has a tendency to promote the formation of secondary dentine or not. I supposed always that the formation of secondary dentine is a natural phenomenon. It is a gradual process of the nerve diminishing and the dentine increasing. I think that is all there is of it. When I attempt to save alive the exposed pulp I use Hill's stopping, pressing it down with a burnisher.

Dr. Taft: Dr. Porter says he thinks the application of this material has nothing to do with the formation of secondary dentine. I am sure he knows better than that.

Apply pepsin to a pulp which is covered over with debris and it operates as an active solvent for debris and removes the diseased portion. The same result follows the application of lactic acid. The pulp is found to be free from the accumulations over it. The action of the pepsin and the lactic acid is perhaps the very same. At least in so far as it operates to remove the debris.

Dr. Porter: There seems to be an idea on the part of some gentlemen in the profession that the application of something over the nerve actually influences nature to deposit secondary dentine. I know very well that if you leave the pulp protected merely with a covering of paper it will not throw out secondary dentine.

Dr. Jennings: Dr. Taft, did you ever have a case of severe periostitis which did not result in the death of the pulp?

Dr. Taft: That complication is of very rare occurrence, but when it does occur the pulp almost always dies.

Dr. Rehwinkle: I have now a case under treatment in which the pulp of a central incisor was destroyed through the influence of an abcess in the lateral incisor. The central looked healthy but the lateral had the appearance of being dead. It had in it a large gold filling. I remarked, this tooth (the central) does not look as if the nerve in it was dead. The other one does. Are you not mistaken? He said he thought not. To satisfy him I drilled through the lingual surface very carefully and found the nerve alive. I then took out the filling of the other tooth and found the nerve not only dead but sloughing. It now became necessary to protect the nerve in the central which had been tapped. I capped it. I then treated the lateral and got it ready to fill. The gentleman claimed all this time that the central was troubling him. I finally retapped it and found the pulp dead and discharging. I am satisfied that the trouble had been communicated from the diseased tooth to the healthy one.

Adjourned until Evening.

WEDNESDAY, DEC. 2D., 1874.—EVENING SESSION.

The second subject, "Contour Fillings," taken up.

Dr. Bowman: I am in the habit of making a great many contour fillings. Consider them very useful.

Frequently in the case of incisors which have been broken off, we can build up and restore the contour, and preserve the facility of speaking.

I will speak more particularly of the material to be used in effecting this object and of the manner of using it. I have been for quite a while using No. 60 foil. I do not know whether or not it is a fancy, but I think from what my judgment and experience have demonstrated, that I can do better work with it than with the lighter foils. I also find that I can work it more readily than I can the heavier numbers. No. 120 is a little more rigid under the instrument, but I can manipulate No. 60 about as readily as Nos. 5 or 6.

The President, Dr. Smith, here cautioned the gentlemen to adhere to the subject announced for discussion.

Dr. Bowman: I consider it far more advisable to restore the contour in the case of a broken tooth, than to cut it off for the purpose of fitting a pivot tooth, or to extract it. The appearance of the gold is to many eyes unsightly, but considering the superiority of the operation over other operations which I have alluded to, I think that objection should be overruled. It shows an appreciation of the value of the natural organ on the part of the patient.

Every man in the profession is duty bound to save the natural organ when it is possible.

They are too often extracted.

The patient is enabled to use the tooth to much greater advantage if the full contour is restored, than he could if the filling be extended only over the edge of the cavity.

If a V shape space is left between the teeth the food crowds into it and irritates the gum.

Dr. Keely: In many cases broken down teeth can be restored to their former shape and made permanently useful. Of course the permanence of the operation depends upon the quality of material of which the tooth is composed. That is the highest art in our profession—we make the tooth useful, if not for a lifetime, at least for many years.

I recently saw a contour filling which was inserted by a New York dentist—his first attempt at a contour filling. It has done good service for ten years. It was filled with sponge gold. Is now in good state of preservation. I examined it critically with a powerful magnifying glass and found this to be so. The practice of making contour fillings should be encouraged, because the operator is often enabled to lengthen a broken off tooth indefinitely, so as to restore its antagonism with the tooth in the opposite jaw.

Dr. Hunter: It is only lately that I have been giving attention to contour fillings. I believe there are many cases where operations of this character can be made to answer a very good purpose. One of the great causes of failure, I think, is the attempting to do too much. One cause of decay with which we have to contend arises from the shape of the tooth. When decay takes place on the approximal surface of a tooth, it is due to the shape of the tooth. If you in that case restore the contour of the tooth you do nothing to prevent the recurrence of decay. There have been hundreds of contour fillings made which have failed in three or four years, because of decay occurring again either above or below the filling. Where there is plenty of room—where a tooth has been lost, there by all means restore the contour of the tooth adjoining the space, if it be decayed.

Dr. Wells: I would like to ask Dr. Taft a question as to the practicability of restoring the contour of teeth which are worn down to the gums—either upper or lower incisors and cuspids. The nerve living but nearly exposed. I know of a number of such cases.

Dr. Taft: I can not answer that question very definitely without seeing the case. There are cases where it would not be advisable to attempt restoration. You must be governed altogether by circumstances. For instance, in a case where all or nearly all the teeth are standing in the mouth, and the superior and posterior teeth have all worn down almost to the gum. It would not be advisable to restore the contour of the anterior teeth and leave the molars unfilled. You must preserve the antagonism. Where the wearing away progresses slowly and the person experiences no special inconvenience, and it would be necessary to operate upon all the teeth, I should hesitate very much, but if the anterior teeth only are remaining they may frequently be built up. I have in a number of cases built up six, eight, or ten anterior teeth in each jaw thus throwing the jaws apart considerably and changing the articulation. This I have done in the cases of the upper and lower anterior teeth. But where the molar teeth remain it is a matter of very doubtful propriety, perhaps might not even be practicable. But I have built blocks or caps of gold in some cases upon six or eight anterior teeth and two or three molars. Judgment must be used in selecting cases. Many teeth are so frail that they will not bear the introduction of the gold. Again, teeth that are devitalized are often so deteriorated in quality or structure, that if a large gold filling, restoring the masticating surface, be introduced, the tooth afterward, under the strain of mastication breaks away and the filling is lost.

Dr. Taft alluded to a case in his own personal experience. Had a large contour filling inserted fifteen years ago in a tooth from which the pulp had been removed. The substance of the tooth so deteriorated that one wall recently broke away from the filling which still remained in position.

He continued: "Such will oftentimes be the condition of a tooth that a simple filling will last much longer than a contour filling. Aside from the mere question of expediency, there are objections urged by some people to the conspicuous appearance of contour fillings in the anterior teeth.

Dr. Taft then cited another case in his practice, of a gentleman whose upper teeth were quite worn away, even to the gums, and the lower teeth were loose; gums sore and painful. Used screws in the upper teeth and built on caps so as to throw the jaws apart. This relieved the soreness of the teeth above and below. None of the teeth were decayed. The condition of the gums also began immediately to improve.

Subsequently the lower teeth were built up in similar manner.

There then arose two causes of complaint. One was the peculiar, unnatural feeling observed by the patient upon occlusion of the teeth, a feeling which he pronounced to be so disagreeable that he thought he could never become accustomed to it. He was constantly sensible of the *metallic* feeling of the caps.

This impression gradually wore away. Afterward he complained of a burning sensation in the sockets of several teeth. This was relieved by dressing down the surface of the fillings a trifle where he complained of the trouble. Changes of the weather affect him greatly. This patient was of strong constitution; age sixty-seven.

Dr. Butler: It does not seem necessary to me to discuss the propriety of making contour fillings. I am aware that there has arisen, within the last year or two, a diversity of opinion in consequence of some new ideas.

Some parties ignore this modern mode of operating.

Dr. Taft has presented some of the advantages and disadvantages of contour fillings.

I think it is perhaps well to make some distinction between simple contour fillings and large fillings built up to restore a lost crown in restricting the use of the term to the former class.

In the case of decay on the approximate surface, for instance, where we restore half or any portion of the crown, I should call that restoration.

In many cases the spaces caused by decay upon an approximate surface are very annoying to the patient, and there is no way to relieve this excepting by making a contour filling. Much judgment is required to enable us to decide when to attempt restoration and when *not* to do it. We have seen cases of exostosis result from building up a tooth so as to bring undue pressure upon it in the act of masticating. We should bear in mind the fact that gold is an excellent conductor of heat, and might in some cases, be the means of exciting periosteal trouble. The trouble experienced in cases where teeth have been lengthened by filling, even the annoyance caused by occlusion and the peculiar dragging sensation caused by the contact of the fillings, is very similar to the difficulty which most persons have to contend with in becoming accustomed to a set of artificial teeth.

We could hardly conceive that a man sixty-seven years old could become accustomed to this difficulty. The trouble comes from a change of the working of the muscles. In attempting fillings of this class we should recognize the great importance of a firm anchorage, the shape of the cavity, etc., to insure a successful operation. If the walls of the cavity are thin they frequently break away after the tooth is filled. In the case of pulpless teeth, time works a destruction which is almost inevitable. Especially is this the case in young subjects.

Dr. Rehwinkle: The matter of contour fillings is one which is much abused. At the last joint meeting of the Mississippi Valley Association and the Missouri State Dental Society, there was represented on a blackboard an operation which had been performed in Connecticut. It was substantially a case of gold teeth. It showed that a great amount of patient labor and skill had been employed, but I question the taste of the operator who did it.

It is a peculiarity of Americans that they like a display of gold in their teeth. If you go to any foreign country you will see the Americans pointed out as the people who wear gold in their teeth.

This taste for such display should be discouraged, we should be the educators to lead the people to correct views of the object of dental operations. If a man can weld gold into a cavity at all, it is only a question of time and patience how much he can add to it, and how far he can extend the filling. To encourage this class of operations merely for the opportunity it affords the operator to display his skill, is unworthy of any man who respects himself and his profession. In the case mentioned it would have been better in my judgment to have extracted the teeth if they could not have been fitted with porcelain facing or pivoted. That would have been more satisfactory to the patient and would have been in better taste.

But as to contour fillings, generally speaking, I approve of them. We must avoid extremes. In the case of bicuspids decayed on the approximate surface I would endeavor to preserve their shape as far as possible. The fact that a space left between these teeth affords lodgment for the food, is not in my opinion so great an objection. I expect my patients to clean their teeth. If the annoyance of the food pressing into the space will lead them to clean their teeth so much the better. A great deal more harm will result from restoring the contour in many such cases, than would result from leaving the wedge shaped space, especially if the operator neglects to notice how the antagonizing tooth strikes.

If the cusps of a bicuspid tooth in particular be built up too high, the antagonizing tooth may strike against the filling and it then has a tendency to force it out. It will not only force it out but it will frequently break the walls of the cavity away at the same time. In making operations of this kind, my practice is to first fill the cavity with adhesive wax or softened gutta-percha, and by means of it get the exact "bite" of the antagonizing tooth. After finishing the filling it may be necessary to grind it down some with the corundum wheel.

The filling should be inserted in such a way that the strain upon it in masticating will not tend to force it out.

But again, I declare that I can not become reconciled to the idea of turning the mouth into a jeweller's shop. The perfection of art here as elsewhere, is in concealing art. The great object is to restore the tooth to usefulness.

Dr. Clayton: One of the causes of failure is the liability which Dr. Rehwinkle mentioned, of biting out the filling. We must take into consideration the laws of forces in mechanics, when making a contour filling, and learn to judge correctly of the amount of pressure which the filling will bear. Let the point next to the cervical wall represent the fulcrum, and the retaining points the weight to be raised. The force is applied at the distal surface of the tooth—the extremity of the filling. We have here an example of a lever of the second class. In making contour fillings in molars, I care not how strong the walls may be; I want good anchorage on the grinding surface. I would prepare the cavity by cutting across the tooth, and then drilling a hole at the end of the groove thus cut out. Then I have the weight and power very near the same point, so that it is almost impossible to force the filling out.

Dr. French: I am more inclined to speak of the causes of failure in contour fillings than of other points which have been considered, I think that failure is more frequently attributable to errors of judgment in preparing the cavity than to anything else. The anxiety of some operators, lest they should make a conspicuous filling, may lead them to avoid cutting away weakened enamel, which should be removed.

The cervical portion of the cavity is also often slighted. I have had opportunity to examine the fillings in cuspids, by many who are esteemed fine operators. Upon testing them with an excavator, have found them frequently defective at this point. This is the consequence of neglecting to cut down to a solid foundation.

As to retaining points I am utterly opposed to them, I believe in giving the cavity a retaining shape. We can all see the advantage of carrying the gold over the edges of the tooth.

Dr. Taft: The cases of failure are the result of not observing requirements in making the operation. If one fails in any particular in the successive steps of an operation, the result will probably be failure sooner or later. One cause of failure in many instances, arises from the physical incapacity or indisposition of the operator. We are not always in like good condition for undertaking difficult cases. Many times we attempt to operate when we should not.

Again: I think you will find that the very best operators are those who feel that they have the confidence and sympathy of the profession. But if one feels that the profession is looking askance at him and criticising his efforts, he can not proceed earnestly and heartily to do his very best.

Much, also, depends on the disposition of the patient to appreciate his efforts. If you are assured of his appreciation, it acts as a stimulus to impel you to do your utmost to accomplish the desired result.

Dr. Buffett: Many, I have no doubt, are making contour fillings more for the reputation which they expect to gain in the profession, than from considerations of what will be for the best good of their patients. The latter should be the main consideration. Oftentimes the prolonging of an operation unnecessarily for the sake of making the filling a contour, results in irritation and local disturbance, which might have been avoided had the operation been shortened. Patients are often made sick by a tedious operation. The loss of the tooth would have been more willingly endured than the cost in suffering at the time and afterward, of having a large contour filling inserted. As you know, many of these operations require for their performance from three to five hours. Extracting the tooth would be in many instances better for the patient.

Contour filling may be attempts at full restoration or only partial restoration.

I do not think there is one case in fifty where it is wise to attempt to restore the exact original shape of a tooth. Its mal-position in the mouth often precludes the possibility almost of doing it. The main object should be to make it serviceable. Give it such a shape that it will be self-cleansing. We should not make our patients walking advertisements. As a rule, I do not think that when the pulp of a tooth is dead, a contour filling is likely to be durable. I should make contour fillings for men more frequently than for women, because they are not so conspicuous.

Dr. Watt: Let us hear from those who do not favor contour fillings at all.

Dr. Whinnery: My conviction for some time has been that contour fillings, as they are made, have been proven a failure; that is to say, many of them. Fill the central incisors with soft gold, as we used to do twenty-five years ago, and you have seen them saved, while the same operation, performed with cohesive gold, has proved a failure.

Dr. Jennings: I would like to ask the opinion of the members as to the method to be employed in a case of this kind: The tooth is a second superior molar for instance, very firm and solid, there is a cavity at the margin of the gum on the approximal surface. You can not separate the teeth with a wedge on account of their firmness. How shall we reach the cavity to fill it?

Dr. Keely: Cut away until you have sufficient space.

Dr. Jennings: I have come to the conclusion that it may be possible to gain space by wedging.

Dr. Taft: I would cut through from the buccal surface or else cut a slot down through the crown.

Dr. Bowman: I will explain my method: In the first place, I put a thin piece of rubber between the teeth, and when that has effected a slight separation I force in cotton, and instruct the patient to force in more from day to day, as it may be required. You would be astonished to see what can be accomplished in the way of separating the teeth by this method. I know a lady who used white wax to close up and hide a space between two teeth. They were thus separated for fourteen years, wide enough to admit a central incisor between them.

After the teeth were filled, the space was closed up in a few weeks as the teeth settled together.

Dr. Keeley: We must remember the occlusion of the teeth is sometimes such that we can not get a space unless we move several or even all the teeth.

Dr. Butler: The question naturally arises whether it is wise to make a space between the teeth in these difficult cases. The tooth is supposed to be decayed upon the approximal surface. Why has it decayed? Because there has not been sufficient care taken to keep the space clean. Now is it desirable to widen the space and fill the cavity, even if it be done ever so carefully. Would it not be better to cut from inside?

Dr. Watt: I believe that many failures in making contour fillings, compound fillings, occur for the lack of a knowledge of the general principles of civil engineering. The amount of force that is to be brought to bear upon the filling; the direction in which it is to be applied, the resisting power of the tooth; all the considerations pertain to civil engineering.

We labor under a disadvantage. The architect usually knows the relative strength of oak, ash, pine, poplar, etc. It is a more difficult matter to estimate the relative strength of teeth.

While we talk here about the collateral sciences, I believe we have made a mistake in not regarding civil engineering as one of the collateral sciences of dentistry. I wish that all dentists might have the opportunity of taking a course in civil engineering.

Dr. Horton: We can not always make contour fillings when we think it desirable to do so, because the patient objects to it. I rarely advise an operation of the kind when there seems to be a tendency to the formation of abscess, or where the condition of the patient's system is such as to discourage a prolonged and tedious operation. I have for a few years past advised the substitution of an artificial crown in many cases, instead of attempting a contour filling. Nevertheless, when conditions are favorable—when the pulp is alive, and I think all the circumstances warrant it, I *do* resort to contour filling.

This is a matter which has received my most careful con sideration. There are patients for whom I would refuse to operate because they are not willing to let me follow my own methods.

THURSDAY, DECEMBER 3d, MORNING SESSION.

Subject for discussion: "Practical views and results of experience in the use of the various preparations of gold for filling teeth."

Dr. Taft: Comparatively few operators now use only non-cohesive gold, and insist that it is the only suitable form of gold for the purpose. There are some—this was especially true a few years ago—who have assumed that cohesive gold *only* is suitable for the purpose of filling teeth. Still, others again have used both, as circumstances have seemed to indicate. I think that the true course. There are objections to gold in all the forms in which it has been presented. A filling of non-cohesive gold can not be welded into a solid mass. The contour of a tooth can not be restored. The terms *non-cohesive* and *soft* foil are generally considered synonomous.

They are not always the same, for instance, gold that is soft and yielding will often weld readily. But some soft foils will not weld at all, or at least but very imperfectly even after annealing.

Fillings of non-cohesive foil are very liable to be dislodged unless great care is taken to secure them. The use of screws renders the use of non-cohesive foil far more practicable than formerly. This form of gold recommends itself for the reason that it is more readily adapted to the sides of the cavity than the cohesive. It is said that more of it can be packed into a cavity than of cohesive gold. That depends upon the manner in which the gold is manipulated. Introduce a piece of cohesive gold, make pressure upon it, it yields in the direction in which the pressure is made and conforms to the surface. After that is done, in so far as it moves, it leaves a space behind it. That space must subsequently be filled or the plug is imperfect. Cohesive gold is susceptible of being built up into any desired shape. Skillful manipulation is of course requsite to enable any one to adapt it to any inequality of surface sufficiently well to exclude foreign substances from the cavity.

In judging of the comparative merits of the different preparations of gold, we must, in citing cases in our practice, take the aggregate of the cases from which to make our deductions. No correct conclusion can be reached by selecting special instances of success or failure, without regard to the general average. There are many excellent qualities to be credited to each form of gold, cohesive and non-cohesive. We should not disparage either; we should be able to avail ourselves of the best properties of both.

Dr. Whitney: It is my misfortune to be located at Honolulu, where my predecessor, twenty-three years ago, commenced practice, using non-cohesive gold. Under his instruction I had, I think, abundant opportunity of seeing what could be accomplished with non-cohesive foil. After a careful comparison of the two, cohesive and non-cohesive foil, I have reached the conclusion, that as a rule, cohesive gold has accomplished better results than non-cohesive.

In the anterior teeth, I have seen fillings of non-cohesive gold fifteen and twenty years old, in good state of preservation. In the back teeth, on the contrary, the fillings have failed. I think that we should be able to discriminate, and to know when to use the one, and when the other.

Dr. Rehwinkel: In using cohesive gold, I think we are on the right track. Electicisim is the only true course. I am becoming daily more and more convinced, that as much judgment is required in the choice of materials to insure successful operations, as in anything else.

I have never confined myself exclusively to the use of cohesive gold; would not consent to do so. Neither would I consent to use the other exclusively. I make a practice of studying conditions well, and then determine which to use.

Inasmuch as the theory of the operation of cohesive gold has led us within the last five years to use this material to the exclusion of others, I will go farther, and say: that I do not consider the education of any dentist complete, until he is able to manipulate all the materials which present themselves in the operation of filling teeth, whether non-cohesive or cohesive foil or tin. I should insist on every student's knowing how to use these materials.

Many of the advocates of non-cohesive gold seem to forget how many teeth have been lost after being filled with that form of foil. In condemning or praising any one material, we ought not to take isolated cases, but try to arrive at an average of successful or unsuccessful operations. Every one who has used cohesive gold knows the difficulty oftentimes of making a close adaptation of the gold to the walls of the cavity; that is a point which is often overlooked by the young operator.

The use of the mallet has much to do with the success or failure of any operator. We hear the most opposing theories as to the proper weight of the mallet—the material of which it should be made, etc. In my opinion, the important question after all, is as to the malleter. We place half the responsibility and the cause of failure or success in our operations, in the hands of an inexperienced boy. We are often more dependent upon the assistant than we are willing to acknowledge, even to ourselves. Many operators now do their own malleting; I consider that a step in the right direction. We must learn to use both hands equally well.

The dentist must have a variety of resources. We find patients who can not endure our improved appliances; we must be able to adapt ourselves to the circumstances of the case. Here it is that we have an advantage in our ability to manipulate successfully either cohesive or non-cohesive foil. I would not part with my knowledge of the manipulation of soft foil for any consideration; I should be crippled without that knowledge. The manufacturers tell us that they sell more non-cohesive foil now than they did a few years ago. I can explain that : there is always a reaction after going to extremes; there is no doubt at all about it that this question of the use of cohesive foil has been pushed to extremes. There are a great many cases where a combination of the two forms of gold would accomplish better results than the exclusive use of either. Take, for instance, a deep seated approximal cavity. It might be found very difficult to make a perfect operation with cohesive foil alone. I have reached the conclusion after years of thought and experience to use any good material or any method of manipulation which will best accomplish the result I aim at. It will not do to confine ourselves to any one material—to any one mode of practice. We must apply ourselves to investigation, and find out what best suits our individuality. Then failures will be less frequent.

Dr. Butler: The experience of one individual can not go far toward establishing a correct mode of practice, if that individual simply confines himself to a comparison of his own productions and experience with his own. If he goes no farther than that, his judgment is not well founded. We can only know of the value of any mode of practice by comparison among ourselves. Cohesive foil was first introduced about sixteen years ago; from that time to the present, it has been almost universally used. The teachings have been such that men coming into the profession have been inclined to employ cohesive gold to the exclusion of everything else.

It behooves us, gentlemen, to consider carefully whether we are doing the very best we can for those who demand our services.

There has been a great aspiration on the part of the profession, and especially on the part of young men to become operators.

The result is that many have become, not operators, but mere manipulators. They have educated their fingers, but after all, they have not become masters of all the different materials, which, if used more frequently than is commonly, the case would give far better results in many cases.

Some men become very skillful in the use of non-cohesive foil, just as others become skillful in manipulating cohesive foil. We all recognize a fact that thousands of teeth are now filled and preserved which could not have been rendered servicable by the old method of filling.

I remember quite well when a man using cohesive foil and filling successfully large cavities which had been considered beyond all surgery, was called "pirate," and "highwayman," when he charged $5.00 and upward for such operations.

Now, a man who sacrifices such teeth is denounced in terms just as severe. It would be considered mal-practice. These are the results which have followed the introduction of cohesive foil.

Properly used, it is indispensable; but there are many cases where the non-cohesive foil can be used with advantage, since it can be introduced more rapidly and answers the purpose fully as well as the cohesive foil; I will not say better. There are men who make good gold fillings who can not make a tin filling. There are men who put in tin fillings which last from fifteen to twenty years. Cut out one of those tin fillings and the last fragment will cling to the walls of the cavity with the same tenacity you notice in gold.

In attempting to confine ourselves to any one material for fillings, we will often defeat our own ends. We make failures, and we try and try again and again in the same way, and *still* fail, and all the time we are doing ourselves and our patients an injustice.

A more complete knowledge of all these materials is requisite, and if we expect to attain to the standing which we think we deserve, we must investigate. There is a demand for better dentists. The people are becoming enlightened, and the man who would impart his information, must first get his information.

I would much rather operate for the person who appreciates a good operation, than for one who does not discriminate between good or poor operations.

Dr. Bowman: I used soft foil exclusively for from fifteen to twenty years. When attention was first directed to heavy foil, I tried it and became disgusted with it. I thought others did not know what they were recommending. Afterwards I tried it again, and, becoming familiar with its peculiarities, I succeeded in making good operations; I now use it exclusively. I do not pretend to say that others may not make good operations with the light foils; but I am confident in the opinion that I can do better work with the No. 60 gold foil or rolled gold; I fill the nerve canal with No. 60. I think I can carry it more perfectly to the apex of the root; in filling cavities with it you can pack it more closely to the walls of the cavity like tin foil. The lighter gold becomes disintegrated in the using. A heavy foil filling has a different appearance, after it is burnished, from a light foil filling.

I prefer to use one grade of gold because after becoming accustomed to using it, it is better not to change. We are all creatures of habit.

I have not been so well pleased with the heavier Nos.; 120 and upward.

Dr. Jennings: Do you depend upon the spreading qualities of the gold to make a perfect filling?

Dr. Bowman: Not by any means, I depend upon the instrument to pack the gold. But I claim, nevertheless, that the heavy gold spreads better than the light, for the simple reason that in packing the light gold you harden it.

Dr. Jennings: That would be a disadvantage in the case of a frail tooth.

Dr. Bowman: I have found no instance where a filling could be inserted at all, where I could not fill as well against a thin, weak wall, with the heavy as with the light foils.

Dr. Whinery: It has seemed to me for several years, that attention has been too much directed to the use of one particular kind of gold. Individuals, *who do not themselves know* how to use cohesive gold successfully, are impressed with the idea that it ought to be considered unprofessional to use *any other* kind of gold; consequently, they have made much bad work, because of their aspiring to use cohesive gold when they do not really understand the proper mode of using it.

Dr. Horton: I am inclined to advocate the use of non-cohesive foil in many cases; more skill is frequently required for its manipulation than in the use of the cohesive gold.

He spoke of the absolute indispensability of the rubber dam in many cases. He sometimes postpones an operation which he thinks is likely to tax his skill and strength; until he thinks circumstances are more favorable, as, for instance, when he does not feel well himself, or his patient is not in the best condition.

It is important above all thing, to take time for operations. As much or more, depends on the manner in which an operation is performed, and the pains which is taken with it, as on the material used. Some men are enthusiasts on the subject of heavy foils. The dentist must always be master of the situation, and be able to decide what is best to use in any given case. Sometimes he uses the ordinary cohesive foil, unannealed; if it is prepared in too large rolls, it will clog in the cavity. He therefore uses it in strips. Subject passed.

The subject next considered, was the "Status of mechanical dentistry." Sub-divisions as follows:

*a.* Can the mechanical branch be rescued from its demoralized condition and raised to a higher and professional standing?

*b.* The best means for the accomplishment of this.

*c.* Has the time arrived for the separation of the operative or surgical from the mechanical department?

*d.* Advantages and disadvantages of such separation to each department.

Dr. Porter: I am one of the very few perhaps, in Ohio, who ignore wholly the mechanical branch in my practice. It has seemed practicable to separate the two departments in the larger cities, but whether or not it could be done in a country practice, has been a question. I made the change in the Spring of '71; it was predicted by some that I would find myself unable to retain the patronage in the operative department.

I found, however, that the first year after making the change, my operative department increased about one-third. It has never since decreased.

I have not made a set of teeth since December, '70. I know from experience, that a man can demand better fees who adopts operating as a specialty.

Dr. Rehwinkel: When we speak of dentistry as a profession, we are very apt to make no mention in the connection of the subject of mechanical dentistry. The committees in our societies have generally either failed to make a report on the subject or if a report has been submitted, it has been received with so little interest and attention as to discourage any future presentation of the subject. While we boast, and justly, of the many improvements which have been made in practice, in the operative and surgical departments, we have nothing to tell of improvements in the mechanical department. While the one department has advanced, the other has, undoubtedly, retrograded. This is a cause of alarm to many of our best men. The fear, and I think with reason, that by at once withdrawing their interest and attention from mechanical dentistry, it would immediately sink to the level of a mere trade. I think the problem which is presented, of separating the two departments, must necessarily be left to its own solution

There are difficulties in the way of the accomplishment of the object, which I have been unable to see any way to surmount.

Dr. Rehwinkel spoke at some length of the demoralizing influence which has been at work in the department of mechanical dentistry since the introduction of rubber, and which has reduced the status of mechanical dentisty so low, that men in the profession, whose natural taste leads them to prefer operating, have come to neglect the mechanical branch altogether.

The relation of the operative and surgical dentist to the mechanical dentist, has been compared to the relation of the surgeon and the manufacturer of artificial limbs, from which the conclusion is deduced that the two departments are naturally distinct and separate. This is not, however, a necessary conclusion, and, in point of fact, is not sustained by the existing state of things.

If by the separation of the two departments it was understood that the mechanical was to be allowed to extract teeth and perform other operations in the province of dental surgery; in preparing the mouth for the reception of the artificial set, then he was opposed to the contemplated separation.

At all events, he did not consider it within the range or jurisdiction of any society to legislate upon the matter.

He did not feel competent to judge as to whether or not the colleges should drop the subject of mechanical dentistry from their curriculum. The inclinations of students must always be regarded; it is folly to ignore a young man's tendencies, and force him to learn that which is distasteful to him.

At the conclusion of Dr. Rehwinkel's remarks, Dr. Taft moved that the fifth subject be taken up in the connection with the clinic in the afternoon. Carried.

The Society then adjourned.

THURSDAY EVENING SESSION.

Dr. Rehwinkel made remarks bearing on the subject of the decision in equity of the Circuit Court of the Eastern District of Michigan; case of Goodyear Dental Vulcanite Co., et al., *vs.* George Willis. He said: "A year ago we were in hopes that in a case then pending, a decision would be rendered in our favor.

You are by this time apprised of the fact that such expectations have not been realized, that notwithstanding we thought from assurances given us, that we were not without good reason for such hopes; we have been disappointed. It was a great disappointment, and cast a cloud over the profession not only in this State, but elsewhere. Your committee were at a loss what to do; they felt the utmost confidence that the case presented in Massachusetts was as good as could possibly be made out anywhere, that every necessary step had been taken to bring it to a fair presentation before the Court; while they did not see any chance of presenting a plausible case in this State. We had no additional testimony whatever to offer

While that case was in suspense and we were at a loss what to do, a case was commenced in Michigan, upon the decision of which it was expected that several other cases would be decided as by a precedent. The Michigan dentists applied to the profession in the neighboring States for pecuniary assistance; the presentation of that case was fair and encouraging. It had been represented to your committee that important additional testimony was to be offered, by which it was hoped the previous decisions of the Courts would be reversed. We were led to believe so by our counsel; the prospect seemed so fair, that our counsel from this State, Messrs. Cox, Follett & Cochran, expressed themselves as sanguine of success. As a matter of course, our confidence was buoyed up by these expressions, and we were in strong hopes of seeing a result, the reverse of that given in the Massachusetts case. The case was originally set for hearing in June, but owing to the absence of one of the Judges, it was postponed until October. It was then again postponed until November. In the meantime, when the American Dental Association met, the majority of your committee had a personal interview with the gentlemen of the Michigan Association, who were interested in the case, and also with the counsel. Although there was nothing new developed then—the case being precisely as it was during the previous time—still, they were very hopeful, especially the counsel, and reiterated their former statement, that there had been important additional testimony presented, and that the case in Michigan *would be heard upon its merits* As time went on, they failed us time after time in such matters as failing to get their documents completed, etc., and upon consultation with counsel from our own State, your committee saw the propriety and policy of coming to the assistance of the profession in Michigan, because whatever might be the decision in that case, we might expect to abide by the consequences. Upon that principle they have acted; they have done everything in their power to strengthen the hands of our Michigan brethren.

The case was heard in November.

There were some facts presented before that bench which I think it improper here to comment upon. One thing I will mention; after the Honorable Court had heard the argument of one side—or at least in part—the Court decided to pronounce opinion. It was in substance this: that although the patent was by the Court considered invalid, it was not considered proper to differ from the decision of Judge Shepley. So it appears that our friends in Michigan were mistaken as to the force of the testimony. They were also mistaken when they flattered themselves that the case would be heard upon its merits. Dr. Rehwinkel then read a letter from the Ohio counsel, Mess. Cox, Follett & Cochran, touching the matter, which appeared to convey the opinion that the decision rendered was irrevocable and irreversible, and advising those members of the profession in arrears to the Vulcanite Co., to settle on the most favorable terms with them.

He also stated that Mr. S. S. White had authorized him to say that he is $10,500 out of pocket; that he has done all in his power to bring this case to the Supreme Court, that it now rests there, and that if the profession wished to have the case argued further, they will be expected to come forward and contribute of their means.

Dr. Horton read a circular which he had recently received from Mr. Bacon, calling his attention to a list of cases tried in which judgment had been rendered in favor of the Rubber Company. Dr. Rehwinkel then called attention to the question as to what would constitute an infringement in the using of rubber, as the case now stands. He said the profession is now released from all restrictions which were formerly imposed by the Goodyear Company. The using of rubber to attach teeth to a metalic plate does not constitute an infringement; that right has been purchased by the Penna Protective League and presented to the profession. He was not able to say whether the manufacturing of obturators would be called an infringement.

As to using rubber in repairing old plates, he thought the Company would claim their right to impose license for the privilege, but he did not think the claim would be allowed.

Dr. Bowman asked whether or not, in case the Cummings Patent should by the Supreme Court be pronounced invalid, a previous judgment rendered by the District Court against a party, would stand.

Dr. Horton: The decision of the Supreme Court covers all the ground.

Refering to the question which had been asked, as to the liability incurred by anyone using rubber in repairing old plates, he said that point had been settled by the Supreme Court. Hall's Digest gives a case which involves the point, viz.: the right of a license to repair the machine, or the work he had constructed under a license, that license had expired. For instance, a man purchased a license, we will suppose, and it has expired, supposing a plate he had made under the license should fail, he could repair it with impunity. One of the District Courts in New York decided in the case of a man who had repaired a sewing machine under these circumstances, against whom suit was brought; that he should be allowed *to make any part of the machine over again* without paying a royalty.

Dr. Herriott spoke of the fact that the Rubber Company had in some instances made unjust and fraudulent claim against parties who had infringed. He warned the members to avoid being imposed upon.

Dr. Rehwinkel: The question has been asked in regard to the refunding of claims collected of the dentists. If we find it best to settle before a Master Commissioner, can we require him to give us security that the money will be refunded in case the decision of the Supreme Court should be in favor of our profession?

This question will be considered by our counsel, and the result of their deliberation will be announced at the earliest possible moment.

The question then arose as to which would be the best course to pursue, whether to settle with Mr. Bacon, the company's representative, or before a Master Commissioner. Dr. Rehwinkel did not feel justified in expressing an opinion. The President, Dr. Smith, thought the better way would be to settle with the Master Commissioner. He can not assess beyond a legal claim. He will not allow any advantage to be taken.

Thought the profession had nothing to fear in making settlement in this way.

At the request of one of the members, Dr. Smith gave some account of the proceedings in the recent trial in Michigan, at which he was present.

Dr. Bowman then offered the following preamble and resolution: Whereas, the Goodyear Dental Vulcanite Company and Mr. Bacon, in a circular dated December, 1st, 1874, and addressed to the dental profession, state among other things, that "the publisher of the "Dental Cosmos," engaged for several years past in advising a course of litigation, which, for strange reasons, has been followed by a great number of your profession." Resolved, that this Association declares this statement to be contrary to the facts, and testifies that whatever Dr. S. S. White has done in connection with this rubber litigation, has been done by him in the interests and by express solicitation of the dental profession, and we hereby tender him again our thanks. We furthermore request him to continue in this good work, and bring the case now pending before the Supreme Court of the United States, to its final hearing—we pledge him material support.

Dr. Horton spoke of Mr. White's interest in the dental profession, and said he could sympathize with him, and could understand his unwillingness to incur further expense in behalf of its members, unless they should manifest more of a disposition to render pecuniary assistance. Said that when Mr. Fisher had told them that it was cheaper to fight than to run away, less than one hundred dentists out of five hundred in the State, had contributed money to carry on a suit. The consequences which have come upon the profession could have been averted; a compromise could have been made in time past, which would have saved the profession thousands of dollars.

Other members spoke of the obligation which was imposed upon every man in the profession to aid in pushing this matter to a final investigation and decision in the Supreme Court. Adjourned.

FRIDAY, DEC. 4TH., 1874.—MORNING SESSION.

After the transaction of some matters of miscellaneous business, the last subject on the list; dental hygiene, was taken up.

Dr. Taft said he was not certain whether in announcing of this subject, reference was had as to the health of the dentist, or whether it was intended to give the discussion a broader scope and include the care of the teeth by the patient. Various occupations exert a pernicious influence upon health.

Dentistry may be classed among those occupations which require great care on the part of the one who follows the pursuit, to preserve his health.

If the dentist confines himself very closely to his duties his health is likely to suffer. Much depends upon the position assumed in operating; many work in a cramped and unnatural position. This is all a matter of habit and can be avoided if attention is directed to it; many are too much confined to the office, and work too many hours a day. This was manifestly the case a few years ago, when it seemed as though some of the best men in our ranks had injured their health irretrievably. Of late years more attention has been given to this matter, and we are learning the importance of exercise and recreation.

If the preservation of his health is an important matter to the physician, it is still more so to the dentist. Anything which effects the nerves, as loss of sleep or dissipation of any kind, it will readily be admitted unfits the dentist for the proper performance of his duties. How can a man in such condition investigate and diagnose, and operate with justice to himself or his patient?

The dentist, like all others who follow in-doors occupations, should have a diversity of employment; let him divert his mind by study; let him seek relaxation in horticulture or any light, pleasant occupation which will keep him much out of doors. Some have been much benefited by attending the gymnasium.

This matter of health of the dentist deserves far more consideration than it usually receives. Hundreds of men break down every year, by ignoring the simpliest laws of health.

Dr. Horton said he is in the habit of operating as much as possible in the open air; keeps his rooms well ventilated by leaving doors and windows open as much as possible, when the weather will permit; allows the fire to go out at night and re-kindles it in the morning. He thinks a man loses nothing by taking his dog and gun and going out frequently for a long tramp. It is a short-sighted policy which makes a man afraid to leave his office occasionally, lest he should miss a patient. Health is wealth after all. He thinks that what time a man loses from his business and devotes to the cultivation of his physical and mental well being, will be more than made up by the years which he is thereby adding to his lease of life.

Dr. Rehwinkel and others offered remarks upon the subject, indorsing the views which had been presented.

The Society then adjourned.

